# Lifestyle and Health Changes During and After the COVID-19 Pandemic: Insights from This Special Issue

**DOI:** 10.3390/nu17213370

**Published:** 2025-10-27

**Authors:** Vilma Kriaučionienė

**Affiliations:** 1 Health Research Institute, Faculty of Public Health, Lithuanian University of Health Sciences, Tilžės Str. 18, 47181 Kaunas, Lithuania; vilma.kriaucioniene@lsmu.lt; 2 Department of Preventive Medicine, Faculty of Public Health, Lithuanian University of Health Sciences, Tilžės Str. 18, 47181 Kaunas, Lithuania

## 1. Introduction

The COVID-19 pandemic profoundly disrupted everyday life and reshaped lifestyle behaviours worldwide. Numerous studies documented significant changes in diet, physical activity, sleep, and mental health, with long-term implications for well-being. Stress and uncertainty were consistently linked to poorer dietary quality, reduced sleep, and greater use of alcohol and stimulants [1,2]. Food insecurity became a major issue, contributing to inadequate fruit and vegetable consumption [3].

Dietary responses were heterogeneous. While some people improved their eating habits, many reported increased snacking and weight gain associated with sedentary behaviours [4]. Changes in food preferences often reflected coping strategies during restrictions [5]. Excess body weight was also identified as a risk factor for more severe COVID-19 outcomes [6].

Physical activity decreased in large parts of the population, and sedentary routines became widespread, although some individuals managed to sustain healthier habits [7,8]. Sleep disturbances emerged as one of the most frequent outcomes, with around 40% of people affected during the pandemic [9]. Even after restrictions ended, high stress levels persisted, undermining mental well-being [7,10].

Mental health impacts were substantial. Social isolation, reduced resilience, and unhealthy coping behaviours contributed to higher rates of anxiety and depression [11,12]. Research also showed that adopting healthy lifestyle habits could mitigate psychological distress and support recovery [13]. Among older adults, frailty progression, poorer diet quality, and loneliness further highlighted the lasting vulnerability of certain groups [14].

According to the literature, although some healthy behaviours recovered after the pandemic, major challenges in diet, physical activity, sleep, and mental health persisted during and after the pandemic. The next section of this Special Issue brings together diverse manuscripts on lifestyle and health during and after COVID-19, highlighting their main contributions and offering findings that enrich our understanding of lifestyle and health changes during and after the COVID-19 pandemic.

## 2. An Overview of Published Articles and Themes

### 2.1. Diet, Weight, Digital Health, Stress, and Inequalities

Weight gain was one of the most frequently reported consequences of lifestyle disruption during the pandemic. The systematic review by Silva-Lalucci et al. showed that overweight and/or obesity in adults were risk factors for worse COVID-19 outcomes, as well as for the need for intensive care, respiratory support, mortality, and changes in significant blood markers [contribution 1]. In Poland, irregular daily routines, poor diet quality, and reduced physical activity were linked to BMI increases, with gender differences: women were more prone to snack and sweets-centered weight gain. In contrast, men’s BMI increases were related to higher alcohol consumption [contribution 2]. In Danish adults, post-lockdown dietary shifts were heterogeneous: decreases in saturated fat intake coincided with declines in consumption of whole grains and fish and increases in red meat intake, yet weight gain remained a persistent issue [contribution 3].

In Brazil, adults maintaining regular physical activity and moderate food intake during lockdown offered protection against very high stress, whereas inactivity significantly increased the likelihood of anxiety and depression [contribution 4].

The pandemic also underscored the growing role of digital health. Brazilian nutrition-and-diet apps increasingly featured educational content, recipes, and physical activity guidance, highlighting the role of digital tools in health promotion and behaviour change [contribution 5].

Socioeconomic factors further shaped outcomes. A Lithuanian study revealed that adults aged 20–64 with higher education were more likely than those with lower education to increase healthy food consumption and decrease unhealthy food consumption, as well as increase physical activity during the quarantine. The urban residents reported unfavourable changes in nutrition habits more often than those living in villages did. Also, highly educated individuals were more likely to sustain healthier diets and weight control post-pandemic [contribution 6].

### 2.2. Younger Populations: Diet, Lifestyle, and Mental Health

Young people were particularly vulnerable. Polish adolescents displayed elevated emotional overeating, particularly among females, those with obesity, and those experiencing weight gain during the pandemic. Emotional overeating was strongly associated with anxiety, sadness, loneliness, and cravings for both sweet and salty foods [contribution 7].

Thai undergraduates showed multiple unhealthy patterns, including high rates of overweight (up to 33.5%), frequent breakfast skipping, reliance on takeaways, increased intake of unhealthy foods, prolonged screen time (over 10 h daily), low physical activity, and irregular sleep [contribution 8].

Among Lithuanian university students, 40.9% who continued to follow unhealthy dietary habits and reduced physical activity maintained increased body weight post-pandemic [contribution 9]. Among Polish students, low engagement in health-promoting behaviours was common; however, maintaining effective emotion regulation and mental resilience was strongly linked to better quality of life across somatic, psychological, social, and environmental domains [contribution 10]. In Italy, post-pandemic increases in coffee and caffeine product consumption among students paralleled rising anxiety and poor sleep quality [contribution 11].

### 2.3. Special Populations Groups and Families

Some groups, however, demonstrated resilience during the pandemic. Among pregnant women in Poland, the pandemic coincided with improvements in diet, preventive behaviours, daily routines such as rest, sleep, activity, and mental well-being [contribution 12]. Family environments also shaped lifestyle outcomes. Among families of childhood cancer survivors in Israel, the pandemic encouraged more frequent shared meals, reflecting positive adaptation, but also increased screen time and parental stress, highlighting the duality of family-level effects [contribution 13].

These contrasting patterns underscore how crisis environments both challenge and reinforce adaptive behaviours across different contexts and demographic groups.

## 3. Knowledge Gaps and Future Directions

Despite these valuable insights, several limitations of studies persist. The majority of studies (all except the study by Baticta and Silva) relied on self-reported, cross-sectional data, limiting causal inference and raising concerns about recall bias. Longitudinal studies using objective measures of diet, activity, and weight are needed to determine whether pandemic-related lifestyle disruptions were temporary or enduring.

Geographically, research was concentrated in Europe and Latin America, with limited evidence from low- and middle-income countries. These populations may have faced greater inequalities in health access and economic resilience. Vulnerable groups such as older adults, people with chronic illnesses, and socioeconomically disadvantaged populations were underrepresented, despite evidence of disproportionate burdens [contributions 4, 6]. Gender differences emerged, with studies suggesting women faced higher risks of poor diet and reduced activity, yet the behavioural, cultural, and biological mechanisms remain unclear and require further study [contributions 6, 7].

While digital tools uptake increased during the pandemic, their long-term efficacy and equity remain unknown [contribution 5]. Mental health’s strong link to lifestyle behaviours necessitates research on causality and intervention effectiveness [contributions 4, 7, 10], including within family systems [contributions 12, 13].

Mental health was linked to lifestyle behaviours, with distress driving overeating, inactivity, and irregular routines [contributions 4, 7, 10]. However, studies that examine mechanisms or test interventions to mitigate these effects are needed.

Future research should employ longitudinal, multicenter approaches with harmonized methodologies to facilitate cross-country comparisons, ensuring that disadvantaged populations are meaningfully included.

Digital health approaches should be tested for sustained impact and accessibility across socioeconomic groups. Finally, research should preserve beneficial adaptations, such as shared family meals and improved health awareness during pregnancy, while addressing harmful changes, including sedentary lifestyles, emotional overeating, and increased stimulant use [contributions 2–4, 6–13].

## 4. Conclusions

The 13 articles in this Special Issue provide an overview of how the COVID-19 pandemic reshaped dietary habits, physical activity, body weight, and other health behaviours. They document adverse effects, such as unhealthy diets, weight gain, and sedentary patterns, alongside positive adaptations, including stronger family engagement and improved health awareness during pregnancy. By synthesizing evidence across diverse populations, these studies emphasize the importance of social, psychological, and contextual factors in shaping responses to crises.
